# Status of Medical Corps of the Army

**Published:** 1874-12

**Authors:** 


We have received a pamphlet, entitled "Petition of the American Medical Association in behalf of the Medical Corps of the Army, with a brief statement of the facts in the case." Following the petition and statement of facts, we find the form of a bill which is to be laid before Congress at the present session for its action. In this bill it is provided: "That hereafter all the officers of the medical corps of the army, now known by law as surgeons and assistant surgeons, shall be designated surgeons, and that they shall have rank and promotion in accordance with length of service as follows, viz: Surgeons of less than five years service shall have the rank, pay and emoluments of first lieutenants of cavalry; surgeons of more than five and less than thirteen years service, the rank, pay and emoluments of captains of cavalry; surgeons of more than thirteen and less than twenty-three years service, the rank, pay and emoluments of majors; surgeons of more than twenty-three and less than thirty years service, the rank, pay and emoluments of lieutenant colonels; and surgeons of thirty years service and upwards, the rank, pay and emoluments of colonels."

The statement of facts shows clearly the justice and equity of the legislation asked for, as well as its necessity in keeping up the efficiency of the army medical corps, and we hope that the Congress will lend a willing ear to the prayer of the petitioners. Those of our readers who have the ear of members of Congress may do useful service by calling their attention to the petition, and impressing them with its justice and importance.

